# Unveiling an ancient biological invasion: molecular analysis of an old European alien, the crested porcupine (*Hystrix cristata*)

**DOI:** 10.1186/1471-2148-9-109

**Published:** 2009-05-18

**Authors:** Emiliano Trucchi, Valerio Sbordoni

**Affiliations:** 1 Department of Biology, Tor Vergata University, 00133 Rome, Italy

## Abstract

**Background:**

Biological invasions can be considered one of the main threats to biodiversity, and the recognition of common ecological and evolutionary features among invaders can help developing a predictive framework to control further invasions. In particular, the analysis of successful invasive species and of their autochthonous source populations by means of genetic, phylogeographic and demographic tools can provide novel insights into the study of biological invasion patterns. Today, long-term dynamics of biological invasions are still poorly understood and need further investigations. Moreover, distribution and molecular data on native populations could contribute to the recognition of common evolutionary features of successful aliens.

**Results:**

We analyzed 2,195 mitochondrial base pairs, including Cytochrome *b*, Control Region and rRNA 12S, in 161 Italian and 27 African specimens and assessed the ancient invasive origin of Italian crested porcupine (*Hystrix cristata*) populations from Tunisia. Molecular coalescent-based Bayesian analyses proposed the Roman Age as a putative timeframe of introduction and suggested a retention of genetic diversity during the early phases of colonization. The characterization of the native African genetic background revealed the existence of two differentiated clades: a Mediterranean group and a Sub-Saharan one. Both standard population genetic and advanced molecular demography tools (Bayesian Skyline Plot) did not evidence a clear genetic signature of the expected increase in population size after introduction. Along with the genetic diversity retention during the bottlenecked steps of introduction, this finding could be better described by hypothesizing a multi-invasion event.

**Conclusion:**

Evidences of the ancient anthropogenic invasive origin of the Italian *Hystrix cristata *populations were clearly shown and the native African genetic background was preliminary described. A more complex pattern than a simple demographic exponential growth from a single propagule seems to have characterized this long-term invasion.

## Background

The widespread introduction of non-native species has long been regarded as one of the major anthropogenic global changes and threats to biodiversity [1,2]. The remarkable economic and ecological costs of biological invasions make the management of invaders one of the leading challenges in conservation biology [3,4]. Standard methodologies implemented in population biology and new statistical tools recently developed in genetic analysis could help elucidate features and patterns relevant to invasive biology [5]. Likewise, a comprehensive investigation of ecology and phylogeography of native source populations may suggest new insights into colonization and rapid evolutionary dynamics of invasive species [6]. The scarcity of genetic data on the native range of successful aliens has recently been highlighted, along with the suggestion that further comparative studies could address these data in order to recognize some common evolutionary features among invaders [7].

Recently, a general warning on the importance of long-term perspective in invasion biology studies has been raised. Indeed, many researches have dealt with the acute phase of an invasion (i.e., the period soon after the introduction event), often lacking an adequate temporal context (see review in [8]). In particular, scientific efforts should be directed to better understand the long-term changes that occur in the environment and community of both invading and invaded species [9]. In order to clarify the patterns and the evolutionary consequences of biological invasions, long-term perspective studies should focus on different aspects of the introduced populations, such as their genetic diversity and structure, population size at introduction (propagule pressure), growth rate and demographic trend [7]. Considering the usefulness of molecular investigations in such retrospective studies [10], in this paper we focus on the analysis of genetic diversity, population structure and demographic trend of a putative ancient European invader, the crested porcupine *Hystrix cristata*.

Three species of the old world porcupines (Hystricidae, Rodentia), morphologically grouped in the *Hystrix *subgenus, occur all over Asia (*H. indica*) and Africa (*H. cristata *and *H. africaeaustralis*). The Cape porcupine (*H. africaeaustralis*) inhabits the South Africa region, from Cape of Good Hope to the borders of Central Africa tropical rainforests, while the crested porcupine (*H. cristata*) lives in North Central Africa, from the Mediterranean coast to Northern Zaire and Tanzania, and in mainland Italy and Sicily [11]. These species are characterized by an extremely wide ecological tolerance: they can be found in arid grassland and semi-desert habitats, in Mediterranean shrub lands and deciduous forests, as well as on the Atlas and Kilimanjaro mountains, up to 3,500 m (a.s.l) of altitude. The origins of the Italian populations of *H. cristata *have long been debated. Based on the timescale of fossil and archaeological records [12] and on historical chronicles (Plinius, *Naturalis Historia*, VIII, 53), it was hypothesized that this species was introduced from Africa, in the late Roman Age, as a game animal. However, such hypothesis has recently been questioned, and a native European origin of the Italian porcupine has been suggested [13]. A recent spread in North and Central-East Italy over the last century has been well documented, and the presence of archaeological and distributional records from areas where the species is now absent has also been recorded [14]. Given a maximum density of 2–4 specimens/Km^2^[15] and the current area of occurrence, the population living in North-Central Italy can be estimated as ca. 80,000–120,000 individuals.

The main aim of this study is to highlight the geographical origin and the history of the Italian *Hystrix cristata *populations by means of genetic analyses, considering the hypothesis of their introduction in recent times. We also investigate the demographic parameters that characterize this putative invasion, on the basis of the genetic diversity and structure of the Italian populations. Moreover, preliminary genetic data on the African native range are shown.

## Results

**Figure 1:**
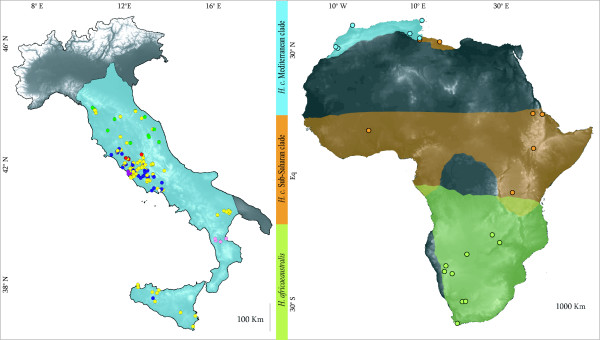
**Samples distribution**. Geographical location of the analyzed samples: 179 *H. cristata *and 9 *H. africaeaustralis *[see Additional file 2]. Occurrence areas of the two major *H. c*. clades and of *H. a*. are coloured (modified from [11]). Colour of Italian samples correspond to haplotype network in Figure 3a.

A total of 2,195 mitochondrial bp, including 726 bp of the cytochrome *b *(cyt *b*) gene, 881 bp of the Control Region (CR) and 588 bp of tRNA-Phe and partial rRNA 12S genes, were successfully sequenced and aligned in 179 individuals of *H. cristata*, 9 of *H. africaeaustralis *and one of *H. indica *(Fig. 1). The summary statistics of the molecular diversity, considering different geographical and phylogenetic partitions, were calculated for the whole mtDNA fragment as well as for each gene, separately [See Additional file 1]. As a whole, 23 segregating sites in *H. africaeaustralis *and 113 in *H. cristata *identify 20 and 9 different haplotypes, respectively. The cyt *b *fragment is characterized by a 36/4 Synonymous/Non-Synonymous substitutions ratio in *H. cristata*, 1/1 in *H. africaeaustralis *and 63/7 in the pooled sample. Considering the whole mtDNA fragment, net genetic distances are: 0.057 (SD 0.002) between *H. cristata *and *H. africaeaustralis*; 0.138 (SD 0.002) between *H. cristata *and *H. indica*; 0.112 (SD 0.01) between *H. africaeaustralis *and *H. indica*. The Italian populations of *H. cristata *have the lowest values for each statistic, with a total of 5 segregating sites and 7 haplotypes, haplotype diversity (H_d_) ranging from 0.28 to 0.62 and average nucleotide diversity per site (π) ranging from 0.0001 to 0.0004. All newly recognized haplotypes have been submitted to GenBank database [Accession Numbers: FJ472530–FJ472546].

**Figure 2:**
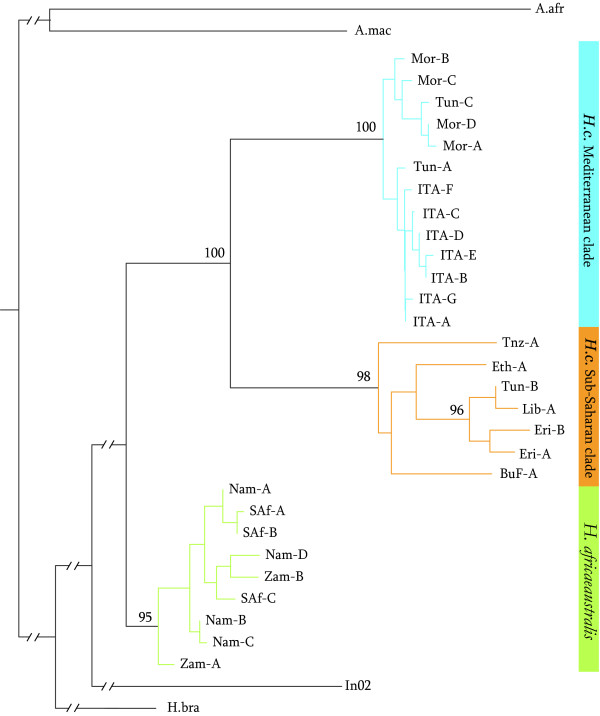
**ML phylogeny**. ML haplotypes tree. Significant node bootstrap support is given. Refer to Additional file 1 for haplotype names; A.afr: *Aterurus africanus*; A.mac: *A. macrurus*; H.bra: *Hystrix brachyurus*.

*Hystrix cristata *haplotypes were organized in two well-supported clades as evidenced by both Maximum Likelihood and Bayesian (data not shown) analyses: a Mediterranean clade, including the samples from Italy, Tunisia and Morocco, and a Sub-Saharan clade, comprising those from East Africa, Burkina Faso, Libya and a Tunisian haplotype (Fig. 2). A clear separation of the two African *Hystrix *species is also supported. Tree topology reveals a close relationship between the Italian samples and Tunisian haplotype Tun-A and an association between this group and the Moroccan sub-clade. Tunisia samples show a particular phylogeographical structure, as the haplotypes from this region were both found in the Moroccan sub-clade and in the Sub-Saharan clade; moreover, Tunisia-Libya samples formed a well-supported group with Eritrean samples. The whole *H. indica *mtDNA fragment and partial rRNA 12S sequences of *H. brachyurus*, *Aterurus macrurus *and *A. africanus *were used as outgroup [GenBank Accession Numbers: AY012117; U12451; AY093658].

**Figure 3:**
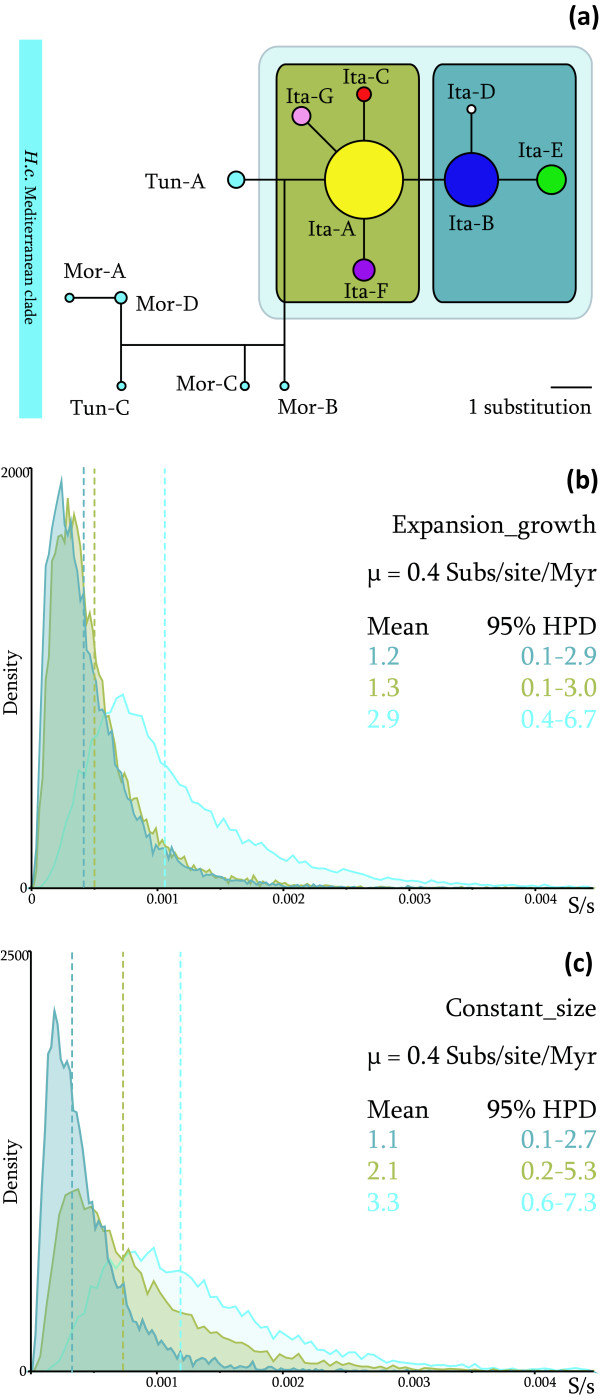
**Mediterranean clade Median-Joining Network and Italian haplotypes timescale**. Median Joining Networks of *H. cristata *Mediterranean clades. Circle size is proportional to sample size (*a*). TMRCAs posterior probability density in Subs/site (S/s) of all introduced haplotypes and of two sub-groups, considering a population size expansion model (*b*) or a constant population size model (*c*); mean and 95% HPD are given in kyr.

Median-Joining Network of the Mediterranean haplotypes is shown in Figure 3a, applying a 0.90% parsimony threshold [16]. In Italy, two common haplotypes (Ita-A and -B) are present in the North-Central and Sicilian populations; three less common haplotypes (Ita-C, -D and -F) were found in Central Italy population, while haplotype Ita-E has a broad area of occurrence in the North-Central region (from 42,356° S to the upper limit of the recent northward colonization front). Another haplotype (Ita-G, 1 substitution in the cyt *b *gene), was sampled only in South Italy. As stated above, Tun-A is the closest relative of the Italian haplotypes and it was found in the North and in the Centre of Tunisia. A close relationship between the other Mediterranean haplotype (Tun-C) sampled in Tunisia and those from Northern Morocco was identified. Here, Mor-B and Mor-C were sampled in West-Central Morocco while Mor-A and Mor-D belong to the Northern Mediterranean coast. Extended branching links, correlated with an extremely wide geographical distribution of the samples, characterize the Sub-Saharan haplotypes median-joining network (data not shown). Global F_ST _of African *H. cristata *native source populations is 0.66 (p = 0).

The timescale of Italian invasion was inferred by means of coalescence-based Bayesian analyses, under two different tree priors which model population size changes through time. Only CR data were used in these analyses. The time to the most recent common ancestor (tMRCA) posterior densities of all introduced haplotypes and of two subgroups are shown in Figure 3b and Figure 3c. Given an intraspecific CR substitution rate of 0.4 subs/site/Myr (see Methods), the root of all samples ranges from 3.35 kyrbp (0.6 – 7.3 kyrbp HPD 95%), under the Cons_s model, to 2.9 kyrbp (0.4 – 6.7 kyrbp HPD 95%), under the Expa_g model.

**Figure 4:**
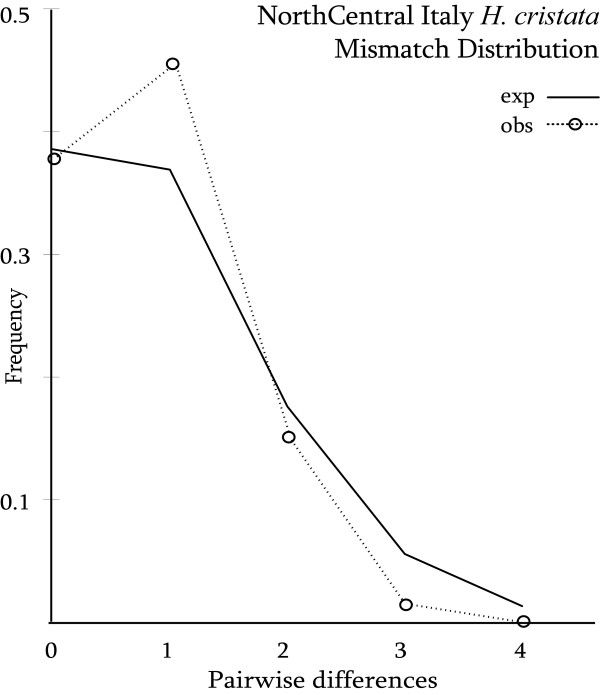
**North Central Italy population MMD**. Mismatch distribution of pairwise differences in CR sequences among North Central Italy samples in comparison with the expected distribution from a non-constant population size model.

North-Central Italy population was used to investigate the demographic parameters that might have characterized the Italian expansion. Neutrality tests did not give significant results (Fu's Fs: -0.815, p: 0.36; R_2_: 0.100, p: 0.62). Similarly, mismatch distribution in the CR sequences (τ = 0.931, time since expansion ≈ 1.8 kyrbp, 1.3 – 2.7 kyrbp according to 95% CR) did not agree with a sudden expansion model, being the sum-of-squared-differences (SSD) statistics equal to 0.01, with p = 0.04 (Fig. 4). Genetic traces of past demographic trend were also inferred in a Bayesian framework. The demographic analysis using the Bayesian Skyline Plot as tree prior (data not shown) did not give significant results. In particular, there was no evidence of demographic expansion of the North-Central Italy *H. cristata *population in the recent past. A Bayes Factor (BF) evaluation between pairs of three analyses, which employed different coalescent tree priors, allowed us to fully reject the Expo_g model (lnBF: Cons_s – Expo_g = 9.7; Expa_g – Expo_g = 9.9), but did not significantly prefer either of the two other models (lnBF Expa_g – Cons_s = 0.117). Indeed, the demographic reconstruction under the Expa_g tree prior suggested a population size increase only in very recent times, after a long period of size stasis post-introduction (data not shown).

## Discussion

Our results confirm the ancient non-native origin of the Italian *H. cristata *populations: *i*) the summary molecular statistics, derived from three mitochondrial genes, highlights the African genetic diversity as being more complex when compared with the Italian; *ii*) the phylogenetic analysis shows the close relationship between the Italian and the Tunisian populations; *iii*) the median-joining network analysis suggests that a Tunisian and the most widespread Italian haplotypes share a common ancestor. These results indicate the Tunisian area as being the most probable source of the Italian introduced populations. In our sample the native geographic range is represented by only five individuals with a global native range FST of 0.66. This supports the assignation of introduced haplotypes to a particular source region with a confidence index of 0.95 (accuracy at regional level of 0.9) as shown by Muirhead *et al*. [17]. The median-joining network analysis also reveals the simple genetic structure of the introduced Italian populations i.e., a single mitogenetic clade of strictly related haplotypes. Both Central-North Italy and the Sicilian populations share the common haplotypes (Ita-A and Ita-B), while Ita-B seems to be absent from southern Italy.

Archaeological records and historical chronicles (Plinius, *Naturalis Historia*, VIII, 53) do not support an introduction of this species in Italy before the late Roman Empire. The first calibrated sub-fossil of *H. cristata *was found in South Italy (Basilicata) and dates back to 1.5 kyrbp [13]. Since we found a close relationship between the Italian and the Tunisian haplotypes, a putative time frame for introduction could span from the early colonization of Sicily by the Phoenicians (ca. 2.5 kyrbp) [18] to the first Italian sub-fossil record. In the light of these considerations, *H. cristata *could have been introduced in Sicily or in mainland Italy during this 1 ky-time frame as a game species, as threatening beasts in circus games or as exotic animals in rich Roman country homes. Considering the coalescent-based Bayesian approach, the time-measured phylogeny of introduced haplotypes partially agrees with this historical framework. However, even if the molecular clock was calibrated using a very fast intraspecific substitution rate [19,20], the mean value of Italian haplotypes tMRCA was dated back to ca. 3 kyrbp, farther than the above hypothesized time span. Conversely, the two recognized Italian subgroups seem to have evolved *in situ*, during the early phases of colonization and spread (2.1–1.1 kyrbp). Nevertheless, this scenario would need to be supported by a rare event of molecular diversity retention in the bottlenecked population during the founding event [21], since genetic drift reduces genetic variation in small populations [22]. Likewise, since Central-North Italy and Sicily share the common Ita-A and Ita-B haplotypes, this rare genetic event during introduction might have occurred in both regions. The possibility of a further acceleration of the molecular substitution rates in extremely recent events as this could also be taken into account [23]. In fact, a time-dependency of the molecular clock rates has been recently proposed and discussed [24,25]. As for *H. cristata *introduction, a substitution rate of 0.5 – 0.6 subs/site/Myr in the mtDNA CR has to be accepted if we consider that the whole Italian molecular diversity was generated *in situ*.

A review of the most recent literature of the demographic parameters that characterize the colonization dynamics does not provide clear evidence of a common pattern during colonization. A successful invasion can take place as a multiple introduction of few individuals from genetically differentiated native populations [26,27], or as a single founder event from extremely bottlenecked gene pools [28]. Being an invasive species, the present population size of Italian *H. cristata *is the result of a demographic and spatial expansion though the genetic signature of this remarkable growth is not revealed by molecular statistics. Although there is evidence of an extremely recent demographic increase, the coalescent-based Bayesian analysis does not clearly support a population expansion model. Since this demographic analysis is based on the assumption of a single panmittic population at mutation/drift equilibrium, the results may be better explained by hypothesizing more than one event of introduction of isolated propagules.

As for the genetic background of *H. cristata *native range, the phylogenetic reconstruction highlighted the existence of two different haplogroups, Sub-Saharan and Mediterranean. The high level of genetic differentiation of the Sub-Saharan clade advocates for a complex evolutionary history, probably related to the broad geographical area of occurrence. Indeed, the haplotypes belonging to this clade were sampled in Central Africa as well as in Tunisia and Libya. The close relationship between the Tun-B/Lib-A and the Eritrean haplotypes suggests the existence of an ecological connection going from East to North Africa, encompassing the Sahara region. This hypothesis is supported by strong evidence of humid phases characterizing North African climate (15-8 kyrbp) [29]. As for the second haplogroup, a low level of genetic differentiation can be detected. This could be the consequence of a less complex evolutionary history or a more recent diversification from a common ancestor when compared with the Sub-Saharan haplogroup. However, the evolution of native range *H. cristata *should be analyzed along with the environmental changes which occurred in Africa over the Late Pleistocene and the Holocene [30]. As widely acknowledged in the Palaearctic Region [31], the distribution and the phylogeographical structure of several African taxa have largely been shaped by these past climatic shifts [32,33]. Further investigation in the North African native range would help to clarify the relationship between the evolutionary history of the African populations and the high invasive potential of the propagules coming from such populations.

## Conclusion

Molecular data strongly evidenced the invasive origin of the Italian *Hystrix cristata *populations from North Africa (Tunisia) introduced during the Roman Age. Although demographic analyses failed to support a population size expansion from a single introduced propagule, they suggest a different scenario in favour of a multi-invasion event. Moreover, this study produced preliminary data on the genetic diversity of native range populations, and provides a useful contribution for further comparative studies on successful aliens.

## Methods

### Samples

A total of 179 *H. cristata *samples (blood and quills) were collected in the field and analyzed between 2006 and 2007: 161 samples came from all over Italy and 18 samples from different African populations in Burkina Faso, Eritrea, Ethiopia Libya, Morocco, Sierra Leone, Tanzania, and Tunisia. Moreover, nine *H. africaeaustralis *samples from Namibia, South Africa and Zambia were analyzed as representatives of the sister taxon species. One *H. indica *specimen from Israel was added in the phylogenetic analyses as outgroup. Location of specimens used in the statistical analyses are given in Figure 1 [see Additional file 2].

### Genetics

Whole genomic DNA was extracted from blood and from modified hairs (quills) of each individual, and the Control Region (CR) sequence was PCR amplified according to Trucchi *et al*. [34]. Two semi-nested primer pairs were developed in order to amplify a 726 bp fragment of cytochrome *b *(CytF1-Cyt06R and Cyt05F-Cyt02R), while the tRNA-Phe and rRNA 12 S partial sequences (588 bp) were amplified using the primer pairs: HyF4-H293 and L82-H618 [35]. All newly designed primers were successfully tested on the three species under investigation. Primer details are given in Table 1. PCR amplifications of *Hystrix *mitochondrial Cyt *b*, and rRNA 12S were carried out applying the following conditions: each 50 μL PCR reaction contained 3–4 μL genomic DNA elution, 100 pmol of each primer, 5 μL 10× PCR buffer, 0.2 mM dNTPs, 1.5 mM MgCl_2_, and 1.5 U Taq DNA polymerase (Eurotaq, EuroClone). PCR thermal profile included: initial pre-heating step at 95°C for 3 min, 35 cycles of 95°C for 30 sec, 56-54°C for 30 sec, 72°C for 30–60 sec, and a 7 min final extension at 72°C. (9700 Thermal Cycler, Applied Biosystem). We did not perform microsatellite analyses since low-quality genetic material, as DNA obtained from our non-invasive sampling strategy, is prone to genotyping errors like allelic dropouts and false alleles identification (rev. in [36]). In our case, the possibility to reduce the errors collecting duplicate samples was not achievable.

**Table 1:** Sequences and references of primers used in this study.

***Gene***	***Primer***	***Sequence (5'-3')***	***Ta °C***	***Ref***.
CR	Hy-For1	CACCATCAGCACCCAAAG	56	[34]
	Hy-For2	AAACCAGCAACCCGACAG	56	[34]
	Hy-For3	AATTGTAGCTGGACTTATAAATC	56	[34]
	Hy-For4	CCCCCGTAAATTTAATAGCTT	56	[34]
	Hy-Rev1	AAGCATTTTCAGTGCTTTRC	56	[34]
	Hy-Rev2	GGGGGTTTGTCAATAGATTG	56	[34]
	Hy-Rev3	GTCCTTCAAGCATTAAAAGAAATC	56	[34]
	Hy-Rev4	AGAAGAGGGATCCCTGTC	56	[34]
	Hy-For5	AATGCAAGACCCCATAAGAC	56	[34]
	Hy-Rev5	GAGTGGGCGATTTTAAGTGT	56	[34]
				
12S	12S-L82	CATAGACACAGAGGTTTGGTCC	54	[35]
	12S-H293	GCACGAGATTTACCAAC	54	[35]
	12S-H618	TATCGATTATAGAACAGGCTCC	54	[35]
				
Cyt *b*	Cyt01F	GAACTAATGACAAACATCCGAAAA	54	This study
	Cyt05F	AACACGATTCTTTGCTTTCCA	54	This study
	Cyt06R	TGGACTAGTACAAGGGCTGTGA	54	This study
	Cyt02R	TTCTGGTTTAATATGGGGAGGA	54	This study

CR: Control Region; 12S: rRNA 12S; Cyt *b*: Cytochrome *b*; Ta: PCR annealing temperature.

### Data analyses

The sequences were edited and aligned using BIOEDIT[37]. Haplotype diversity H_d_, segregating sites S, nucleotide diversity averaged per site π [38] and, Synonymous/Non-Synonymous ratio (as regards cytochrome *b *gene) were calculated using DNASP[39]. The same software was used to describe number and frequency of different haplotypes and to calculate θ per sequence from segregating sites [40], being this diversity value an informative synopsis of the metrics used. Net genetic distances based on the proposed GTR + Γ + I [41] model of sequence evolution were calculated on the whole analyzed mtDNA fragment, exporting tip-to-tip distances obtained from Maximum-Likelihood phylogeny calculated in TREEFINDER[42]. A Maximum-Likelihood (ML) and a Bayesian (Ba) methods were used to assess the phylogenetic relationships among mitochondrial haplotypes. TREEFINDER was used to evaluate the best-fit model of sequence evolution for each analyzed data set, using the Akaike Information Criterion. As regards the whole dataset, the proposed model was a GTR + Γ + I with unequal base frequencies. TREEFINDER was used to build the ML phylogeny, while BEAST[43] was used to reconstruct the Ba phylogeny. Robustness of the ML trees was tested with 1,000 bootstrap replicates. The relationship among the haplotypes belonging to each tree sub-clade was reconstructed by means of a Median-Joining network analysis, as implemented in NETWORK[44].

According to Muirhead *et al*. [17], global Fst among native populations was used to assess the confidence index to classify introduced individuals to a source geographic area. It could prevent incorrect identification of invasion source when a low number of individuals has been sampled in putative source populations. Three putative African sources were considered: Tunisia-Libya, Morocco, and East Africa comprising Eritrean, Ethiopian and Tanzanian samples. A Kimura 2-parameters distance method and 10,000 permutations was used to calculate global Fst with the software ARLEQUIN[45].

TMRCAs of all Italian haplotypes and of 2 sub-clades were estimate on the base of Control Region data using BEAST. Constant_size (Cons_s) and Expansion_growth (Expa_g) tree priors were compared as well as the Relaxed and the Strict molecular clock models. At least three independent runs 3 × 10^6 ^steps long were performed for each dataset. Convergence of chains, effective sample size, estimates and credible intervals for each parameter were analyzed with the program TRACER[43]. TMRCAs were dated using a 0.4 subs/site/Myr substitution rate. This rate has been proposed by Rajabi-Maham *et al*. [19] for intraspecific polymorphism analyses in *Mus musculus domesticus*. Since similar mitochondrial substitution rates were recorded in Hystricidae and Muridae [46], this molecular rate was also considered in our study.

The mtDNA sequences were analyzed in order to find traces of demographic trend from introduction, growth rate and present population size. Several approaches were attempted. These analyses were performed only on the North Central Italy samples, which can be considered, to a large extent, as a panmittic expanding population. Only the fast-evolving mtDNA CR-I was used in these investigations. Two different neutrality tests, which have been shown to be the most powerful [47], were used to investigate for population expansion or bottleneck on the sampled CR-I sequences: Fu's Fs [48] statistics were calculated, as implemented in ARLEQUIN and R_2 _[47] as implemented in DNASP. Significance of Fs was assessed by 10,000 randomization, while significance of R_2 _was determined by means of 10,000 coalescent simulation on the basis of observed number of segregating sites in the sample. The mismatch distribution of pairwise differences between sequences was examined to find genetic traces of the demographic trend. The sum-of-squared-differences (SSD) statistic was used to test the goodness-of-fit between the observed mismatch distribution and that expected under a sudden expansion model. The significance of SSD was assessed by 10,000 parametric bootstrap re-sampling [49], using ARLEQUIN. Past population demography was also investigated with a Bayesian Skyline Plot (BSP) [50], as implemented in BEAST. This coalescent-based approach calculates the effective breeding population size (N_ef_) through time directly from sampled sequence data and not from previously recognized phylogeny. This procedure can therefore account for uncertainty associated with reconstructed phylogeny. The analysis was done using a different time segmentation ranging from 5 to 10 groups (past N_ef _points) and substitution model (HKY + I) [51]. Three independent runs of 50 × 10^6 ^iterations for each grouping scenarios were performed. In addition, separate runs were performed, using different coalescent tree priors which model population size through time: Cons_s, Expa_g and Exponential_growth (Expo_g). Three independent runs of 30 × 10^6 ^iterations for each scenario were done. Convergence of chains, effective sample size, estimates and credible intervals for each parameters and demographic reconstructions were analyzed with the software TRACER. The three different demographic models were then compared to each other in order to assess their relative fit to the data. Comparisons were made under TRACER workspace evaluating the marginal likelihood ratio (marginal with respect to the tree prior) of pairs of models (Bayes Factor calculation). The best approximation to the marginal likelihood comparison was found calculating the Bayes Factor on the tree Likelihood trace [52,53].

## Authors' contributions

ET carried out the molecular analyses, designed the study, performed the statistical analyses and wrote the manuscript. VS conceived and coordinated the study. All authors read and approved the final manuscript.

## Supplementary Material

Additional File 1 **Summary molecular statistics**. Summary statistics of geographic and phylogenetic groups of analyzed samples for Cytochrome *b*, Control Region, rRNA 12S and the whole mtDNA fragment (composite). Click here for file

Additional File 2 **Sample details**. Haplotype ID and locality of analyzed samples (gps point in decimal degrees). Click here for file
